# Variable coordination of cotranscribed genes in *Escherichia coli *following antisense repression

**DOI:** 10.1186/1471-2180-6-97

**Published:** 2006-11-21

**Authors:** Rikard Dryselius, Abbas Nikravesh, Agne Kulyté, Shan Goh, Liam Good

**Affiliations:** 1 Department of Cell and Molecular Biology, Programme for Genomics and Bioinformatics, Karolinska Institutet, Berzelius väg 35, 171 77, Stockholm, Sweden; 2 Department of Bacterial Infections, Research Institute for Microbial Diseases, Osaka University, 3-1, Yamadaoka, Suita City, Osaka, 565-0871, Japan; 3 Life Sciences, Södertörns University College, Alfred Nobels allé 3, 14152 Huddinge, Sweden

## Abstract

**Background:**

A majority of bacterial genes belong to tight clusters and operons, which complicates gene functional studies using conventional knock-out methods. Antisense agents can down-regulate the expression of genes without disrupting the genome because they bind mRNA and block its expression. However, it is unclear how antisense inhibition affects expression from genes that are cotranscribed with the target.

**Results:**

To examine the effects of antisense inhibition on cotranscribed genes, we constructed a plasmid expressing the two reporter genes *gfp *and *DsRed *as one transcriptional unit. Incubation with antisense peptide nucleic acid (PNA) targeted to the mRNA start codon region of either the upstream *gfp *or the downstream *DsRed *gene resulted in a complete expression discoordination from this artificial construct. The same approach was applied to the three cotranscribed genes in the endogenously expressed *lac*-operon (*lacZ*, *Y *and *A*) and partial downstream expression coordination was seen when the *lacZ *start codon was targeted with antisense PNA. Targeting the *lacY *mRNA start codon region showed no effect on the upstream *lacZ *gene expression whereas expression from the downstream *lacA *gene was affected as strongly as the *lacY *gene. Determination of *lacZ *and *lacY *mRNA levels revealed a pattern of reduction that was similar to the Lac-proteins, indicating a relation between translation inhibition and mRNA degradation as a response to antisense PNA treatment.

**Conclusion:**

The results show that antisense mediated repression of genes within operons affect cotranscribed genes to a variable degree. Target transcript stability appears to be closely related to inhibition of translation and presumably depends on translating ribosomes protecting the mRNA from intrinsic decay mechanisms. Therefore, for genes within operons and clusters it is likely that the nature of the target transcript will determine the inhibitory effects on cotranscribed genes. Consequently, no simple and specific methods for expression control of a single gene within polycistronic operons are available, and a thorough understanding of mRNA regulation and stability is required to understand the results from both knock-down and knock-out methods used in bacteria.

## Background

Antisense agents are useful for functional genomics in bacteria as they can down-regulate expression of specific genes by binding mRNA and inhibit translation. As antisense agents may be titrated into cultures of wild type cells they are attractive for studies of any gene, even those stringently required for survival. Also, down-regulation through externally added antisense agents bypasses the need for genome modifications, thereby allowing gene specific studies of both clustered and overlapping genes. A remaining question, however, is whether antisense agents retain specific inhibitory effects when cotranscribed genes are targeted.

Prokaryotic genes are commonly expressed as polycistronic transcripts. For *E. coli*, several concordant studies predict that the >4400 genes are expressed as 2500–2800 transcriptional units (TUs) of which 70–75% are expected to be monocistronic [1-3]. Therefore, over half of the *E. coli *genes are expressed from multigene operons. Similarly, predictions performed on various other prokaryotic genomes show that between 27 and 78% of the genes are expressed within polycistronic transcripts, with an average well above 50% [2]. As a practical consequence, reliable single ORF genetic knock-out techniques are difficult or unavailable for functional genomics studies on a majority of prokaryotic genes.

We are interested in the use of antisense peptide nucleic acid (PNA) for functional genomics as well as antibacterial drug discovery. PNA is a nucleic acid mimic with the phosphodiester backbone replaced with pseudo-peptides and can bind to other nucleic acids with high affinity [4]. PNA targeted specifically to mRNA results in inhibition of gene expression, but unlike many other synthetic nucleic acids PNA does not induce RNase H mediated cleavage [5,6]. Instead, inhibition occurs through steric hindrance of the translation machinery [6], and potent antisense effects can be obtained by targeting the mRNA start codon region [7].

Here we examined the consequences of PNA-mediated inhibition of cotranscribed gene expression in *E. coli*. The separate mRNA start codon regions within both an artificial TU (*gfp*/*DsRed*) and the endogenous *E. coli lac*-operon were targeted by antisense-PNAs followed by protein quantifications for each ORF. Expression from the artificial *gfp*/*DsRed *construct was completely discoordinated, as only GFP levels were reduced when the *gfp *start codon region was targeted, and only RFP levels were reduced when the start codon region of *DsRed *was targeted. However, a directional downstream expression inhibition of the native *lac*-genes was observed. Interestingly, mRNA-level measurements confirmed this expression pattern, although inhibition was less pronounced than at the protein level. The main conclusion is that steric hindrance of translation initiation affects cotranscribed genes to a variable degree. Therefore, simple, specific methods for functional studies on most bacterial genes are lacking.

## Results

### Antisense effects on genes within an artificial operon

**Figure 1:**
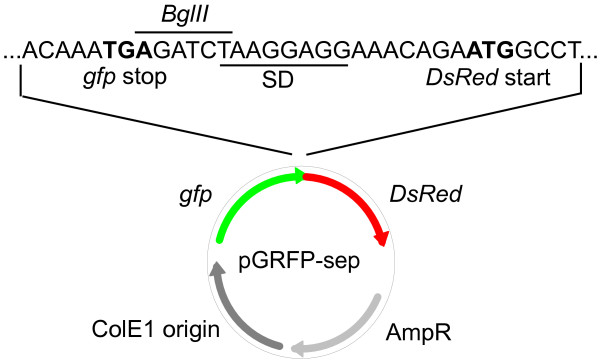
**Illustration of plasmid pGRFP-sep**. The parent green-red fusion plasmid pGRFP [8] was modified by insertion of a *gfp *stop codon, a strong Shine-Dalgarno sequence and start codon for *DsRed*. pGRFP-sep expresses *gfp *and *DsRed *as one transcript encoding two different proteins.

**Figure 2:**
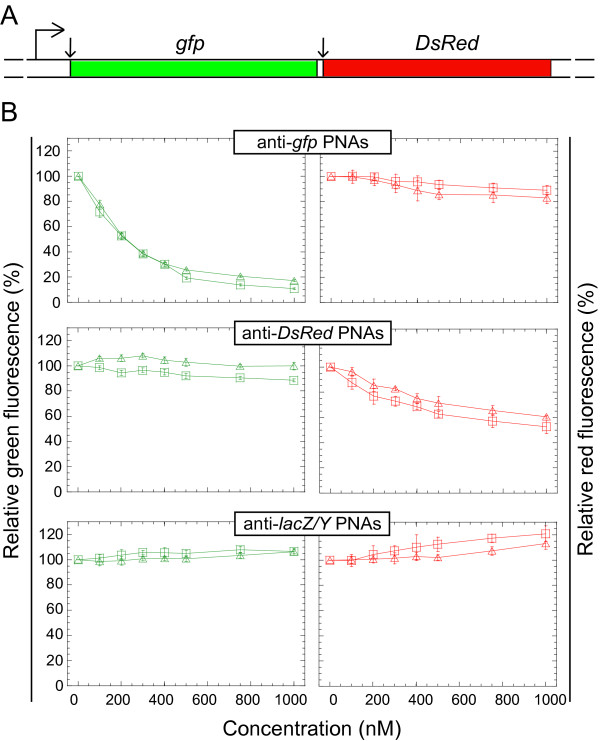
**Expression of GFP and DsRed from an artificial polycistronic transcript after treatment with anti-*gfp *and anti-*DsRed *PNA**. (**A**) Structure of the artificial *gfp*/*DsRed*-operon with the antisense target sites indicated by a small arrows. (**B**) Relative amounts of GFP (left panel) and DsRed (right panel) in cells after treatment with increasing concentrations of anti-*gfp *(top), anti-*DsRed *(center) and controls consisting of anti-*lacZ *and anti-*lacY *PNAs (bottom). For treatment with anti-*gfp *and anti-*DsRed *PNAs, squares and triangles represent values for two different antisense sequences that share genetic targets. For the control experiments, squares represent treatment with anti-*lacZ *PNA and triangles treatment with anti-*lacY *PNA. All data points are derived from six double samples using non-treated cells as standard and pGRFPsep-free cells to remove background fluorescence.

To obtain a system producing polycistronic mRNA transcripts where expression from cotranscribed ORFs is easily quantified we inserted a stop codon between the *gfp *and *DsRed *parts in the pGRFP double reporter plasmid [8]. Also, to improve prospects for translational coordination between the genes, the intergenic distance was kept short and a strong SD sequence was included [9]. The resulting pGRFPsep construct (Figure 1, Figure 2A) was introduced into *E. coli *AS19. After treatment with varying concentrations of anti-*gfp*, anti-*DsRed *or two unrelated control PNAs (anti-*lacZ *and anti-*lacY*, see Table 1 for details) protein expression was determined. As seen in Figure 2B, treatment with the anti-*gfp *PNAs resulted in a reduction of GFP levels, whereas RFP levels remained largely unaffected (upper panel). Similarly, the anti-*DsRed *PNAs only reduced the expression of RFP (Figure 2B, middle panel). Moreover, the control experiments with PNAs directed to unrelated mRNA targets did not inhibit the expression of GFP or DsRed (Figure 2B, lower panel). Therefore, the results show a specific downregulation of expression even though the coding sequences belong to the same transcriptional unit.

### Antisense effects on gene expression within the *lac*-operon

**Figure 3:**
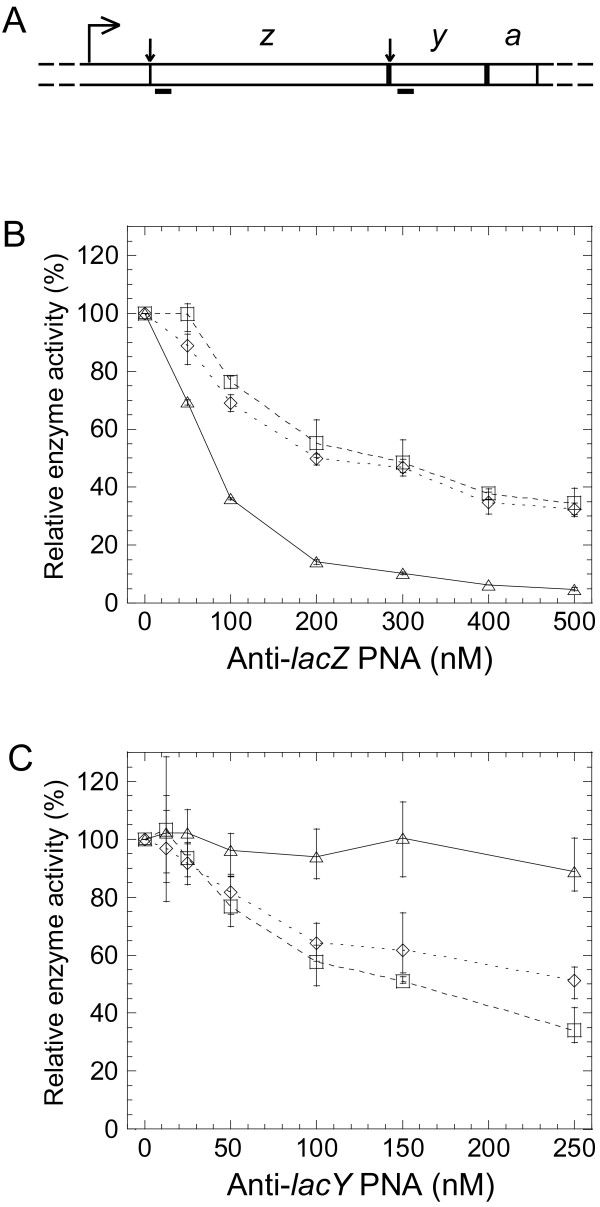
**Protein expression from the *lac*-operon after treatment with antisense PNA directed to *lacZ *and *lacY *mRNA start codon regions**. (**A**) Structure of the *lac*-operon with antisense target sites on the mRNA indicated by small arrows. The solid bars below indicate amplified regions in subsequent qPCR experiments. Relative amounts of LacZ (triangles), LacY (squares) and LacA protein (diamonds) were detected in cultures induced with 100 μM IPTG and treated with increasing concentrations of either anti-*lacZ *(**B**) or anti-*lacY *PNA (**C**). Data points show the mean from triplicate samples where non-treated samples are used as standard and un-induced samples are used to remove noise from the assays.

The above result describes the effect of antisense PNA targeted to cotranscribed genes within a system where translation is discoordinated. However, as this system is an artificial construct lacking naturally evolved regulatory components and sequences we decided to continue the study with an endogenous operon. We targeted the genes in the chromosomally expressed *lac*-operon and determined the effect on the expression of its three structural genes; *lacZ*, *lacY *and *lacA *(see Figure 3A). Treatment with anti-*lacZ *PNA resulted in a strong inhibition of the *lacZ *encoded protein (Figure 3B). Also, expression from the two downstream genes *lacY *and *lacA *was markedly reduced, however not to the same extent as *lacZ *expression. When bacteria were incubated with anti-*lacY *PNA, *lacY *and *lacA *gene expression was reduced, whereas expression from the upstream *lacZ *gene was unaffected (Figure 3C). Unfortunately, we did not obtain any data with anti-*lacA *PNA as this displayed strong growth inhibitory effects. Nevertheless, the results show that antisense targeted to an upstream gene in the *lac*-operon has a large impact on the expression from the downstream genes. In contrast, no upstream coordination was observed, suggesting a directional inhibitory effect in the *lac*-operon.

To ensure that the anti-*lacZ *and anti-*lacY *PNAs did not interfere with bacterial growth and examine whether expression of an unrelated protein was affected by the presence of antisense, we determined growth rate and Lux activity in cells carrying the plasmid pLux1 following treatment with PNAs that target the *lac*-operon. Neither growth nor Lux expression was altered by the PNAs when added in concentrations up to 1000 nM (see Additional file 1).

### Antisense effect on mRNA levels

**Figure 4:**
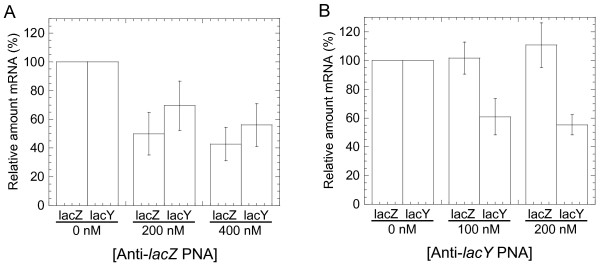
**Relative amounts of *lacZ *and *lacY *mRNA species after antisense PNA treatment**. Levels of specific mRNAs were determined using real time PCR with 16S rRNA as an internal control. Mean delta Ct values (target-rRNA) were compared across concentrations of anti-*lacZ *and anti-*lacY *PNAs using unpaired t-tests assuming equal variance among groups. Significance was set at p < 0.05 and error bars reflect standard deviation. (**A**) Samples from cells treated with 0, 200 and 400 nM concentrations of anti-*lacZ *PNA. (**B**) Samples from cells treated with 0, 100 and 200 nM concentrations of anti-*lacY *PNA.

To better understand the expression pattern observed at the protein level, we used quantitative real time PCR (qPCR) to study the impact of antisense PNA inhibition at the mRNA level. Samples from bacteria treated with varying concentrations of either anti-*lacZ *(0, 200 and 400 nM) or anti-*lacY *PNA (0, 100 and 200 nM) were analysed for their *lacZ *and *lacY *mRNA content (Figure 4). Treatment with anti-*lacZ *PNA significantly reduced *lacZ *mRNA at both 200 nM and 400 nM (p < 0.01 and p < 0.001, respectively) (Figure 4A). *LacY *mRNA was less affected and a significant reduction was only obtained with the higher anti-*lacZ *PNA concentration (p < 0.05). Moreover, there was a significantly higher reduction of *lacZ *mRNA than *lacY *mRNA after treatment with the lower antisense dose (p < 0.05), but not at the higher dose (p = 0.066). Interestingly, a comparison between protein and mRNA levels after anti-*lacZ *PNA treatment reveals a coupled reduction (cf. Figure 3B with 4A). Anti-*lacY *PNA significantly reduced the level of *lacY *mRNA at both 100 and 200 nM concentrations (p < 0.05 and p < 0.005, respectively), whereas *lacZ *mRNA levels were comparable to untreated cells (Figure 4B). Again, a coupled reduction of transcript and protein levels is demonstrated as LacY but not LacZ protein concentrations were reduced by anti-*lacY *PNA (cf. Figure 3C with 4B).

**Figure 5:**
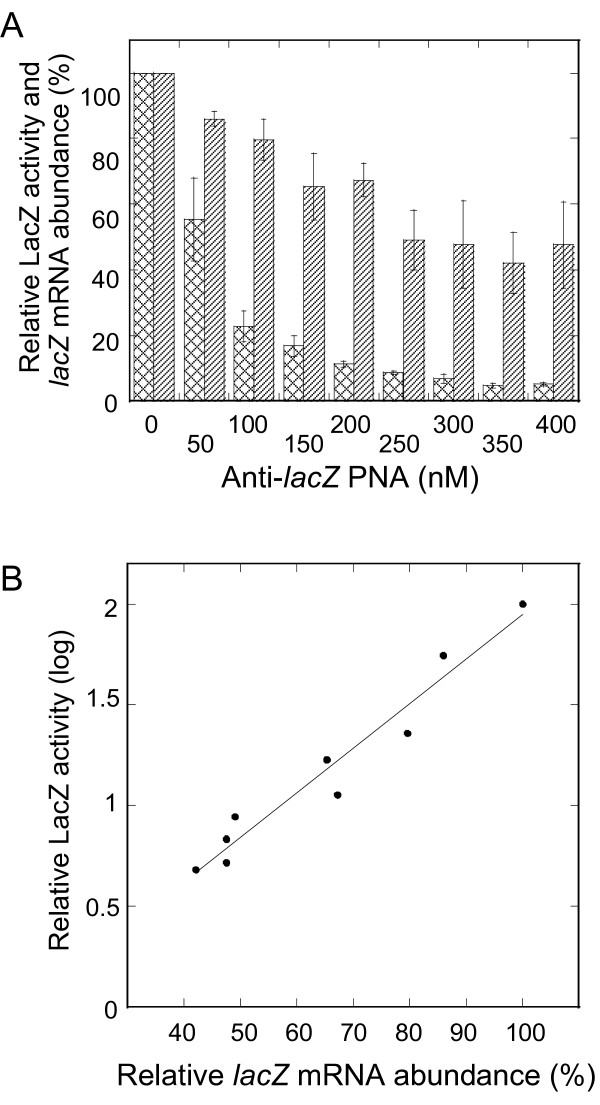
**Dose effect of antisense PNA on LacZ protein activity and *lacZ *mRNA abundance**. AS19 cells induced with 100 μM IPTG and treated with increasing concentrations of anti-*lacZ *PNA were harvested at ΔOD_550 _< 0.1 for LacZ activity and RT-PCR measurements. (**A**) Relative amounts of LacZ protein (hatched bars) and *lacZ *mRNA (slashed bars) are shown as mean values from three separate experiments +/- std. Nontreated samples were used as standard and un-induced samples were used to remove background. (**B**) Nonlinear correlation between LacZ activity and mRNA abundance after treatment with anti-*lacZ *PNA at increasing concentrations. The average values shown in panel (**A**) were plotted following log transformation of the LacZ activity data. Linear regression analysis of the plotted data indicates R = 0.974 for the correlation with the two tailed p < 0.001.

The above results demonstrate a dose dependent gene expression inhibition at both the mRNA and protein levels, with a proportionally stronger reduction of protein. To further examine this relationship we performed a more detailed analysis of *lacZ *gene expression with anti-*lacZ *PNA doses ranging from 0 to 400 nM concentrations at 50 nM intervals. Again, a coupled reduction of both protein and mRNA levels was observed (Figure 5A). Supporting our previous observations (Figure 3B and Figure 4A), increasing doses of anti-*lacZ *PNA resulted in a negative exponential decrease in LacZ protein activity whereas the decrease in *lacZ *mRNA was linear (Figure 5B).

## Discussion

The aim of this study was to examine antisense PNA mediated inhibitory effects when genes in polycistronic operons are targeted. We demonstrate that protein expression from cotranscribed genes can be substantially inhibited by antisense and that there is a stringent coupling between protein and transcript levels when ribosomal binding is hindered.

We first demonstrate that antisense PNAs directed to the mRNA start codon region of either gene in an artificial *gfp*-*DsRed *dual reporter operon lead to a fully discoordinated reduction in gene expression. This was followed by targeting the separate genes in the chromosomally encoded *lac*-operon. Interestingly, in this case a different response to antisense PNA mediated translational inhibition was observed. Targeting the 5' *lacZ *resulted in a stronger inhibition of LacZ than the downstream LacY and LacA proteins, demonstrating a partial discoordination in protein expression, a phenomena previously observed by Pestka and co-workers when "anti-mRNA" directed to *lacZ *mRNA was expressed from a plasmid [10]. When *lacY *was targeted, downstream LacA expression was reduced as much as LacY, whereas LacZ was unaffected. The general differences in expression response between the *gfp*-*DsRed *transcript and the *lac*-operon transcript can likely be assigned to differences in expression regulation: For the naturally occurring *lac*-transcript, and also for many other endogenously expressed polycistronic transcripts, a variety of regulatory mechanisms have evolved to balance gene expression according to the cell's needs. The *gfp*-*DsRed *transcript, however, is not a natural component for bacteria and is therefore not likely to be affected by such specific regulatory mechanisms. Therefore we conclude that antisense PNAs targeted to genes belonging to polycistronic transcripts are very likely to affect cotranscribed genes, thereby rendering gene functional studies on such genes more difficult.

Several studies show that efficient ribosomal binding is necessary for bacterial mRNA stability [11-13], and that actively translating ribosomes protect mRNA from RNase E mediated decay [14]. Also, it has been shown that enhanced transcription rates of *lacZ *mRNA lead to decreased production of LacZ protein and lower transcript stability, probably due to prolonged exposure of unprotected mRNA behind the RNA polymerase [15]. Antisense PNAs inhibit gene expression by steric hindrance of the translation machinery and the effect is most potent when targeting mRNA ribosome binding sites [6,7]. By blocking ribosome loading, antisense PNA may have an effect on mRNA levels. Therefore, we investigated the impact of anti-*lacZ *and anti-*lacY *PNA on mRNA. We observed that the pattern of mRNA levels following antisense treatment was similar to that of protein levels within the *lac*-operon, suggesting that inhibition of translation by antisense PNA indeed has a destabilising effect on mRNA. Destabilisation of target mRNA is in accordance with that observed by Forsyth and co-workers when they used expressed antisense RNA to down-regulate gene expression [16]. Therefore, it appears that the activity of sterically blocking antisense agents, at least in some cases, can be assessed by examining the mRNA levels. The reason for this coupling of mRNA and protein levels is likely due to an increased exposure/decreased protection of the mRNA.

The altered expression pattern of the *lac *genes as a response to antisense treatment raises at least two new questions: (i) "Why is expression from the downstream *lacY *and *lacA *less inhibited than *lacZ *with anti-*lacZ *PNA?" and (ii) "How can *lacZ *expression remain unaffected when cells are treated with anti-*lacY *PNA?" If one only considers protein expression levels it would be possible to explain the partly discoordinated downstream inhibition as being due to noise in the LacY and LacA assays. However, standard curves created with different mixtures of induced and non-induced cells gave linear responses (data not shown). Moreover, mRNA quantification experiments revealed a coupled pattern between transcript and protein levels when antisense was added. An alternative explanation could be that reinitiation at nearby start codons after a ribosome has reached the stop of a coding region is somehow affected in a polar manner. However, it has been demonstrated that reinitiation of ribosomes is a comparatively rare event during translation [9,17], an observation also supported here by our results from the double reporter experiment. It is therefore unlikely that altered reinitiation could explain the rather large expression differences between LacZ and LacYA. Also, the effect on mRNA suggests a different explanation. We believe the explanation may lie in direct inhibition of LacZ protein synthesis together with Rho-dependent premature transcription termination. Cessation of translation induces Rho-dependent termination, probably due to increased exposure of Rho protein binding sites behind the transcribing RNA polymerase [18]. As it has previously been shown that the *lacZ *coding region contains several Rho binding sites [19], antisense inhibition of ribosome binding at the *lacZ *mRNA should lead to increased Rho binding and therefore increased premature transcription termination. However, as translation inhibition of, in this case, LacZ is the primary event; premature transcription termination is expected to be slightly delayed. Indeed, such a tendency can be observed at both protein and mRNA levels following antisense inhibition (Figure 3B and 4A). Moreover, the analysis using a more detailed anti-*lacZ *PNA dose range confirms this pattern (Figure 5): While mRNA amounts decreased linearly with increasing concentrations of anti-*lacZ *PNA the decrease in LacZ protein activity was much more pronounced.

Why, then, is *lacZ *not affected by downstream targeting by the anti-*lacY *PNA? Even though the *lac*-operon is thought to be fully transcribed before being processed [20], it has been observed that *lacZ *transcripts are several folds more abundant than *ZYA *transcripts [20,21]. This imbalance can be explained by RNase E cleavage within the *lacZY *intergenic region that stabilises the *lacZ *portion of the transcript [22], an event that decouples the coding regions of *lacZ *and *lacYA *[23]. According to this model, most of the LacZ product is likely to be translated from processed monocistronic transcripts that can not be affected by antisense binding to *lacY *mRNA.

## Conclusion

In summary, our experiments show that antisense inhibition of genes within polycistronic operons can strongly affect cotranscribed genes. We further show that inhibition is followed by a decreased stability of the transcript that is coupled with translational reduction. This decreased mRNA stability can not be explained by RNase H activity because antisense PNA do not induce such cleavage. Instead, we believe that the presence of antisense unmasks the mRNA from translating ribosomes and thereby increases exposure to RNase cleavage and intrinsic decay mechanisms that regulate RNA stability. A practical issue associated with the stringent correlation between transcript and protein levels is that mRNA quantification may provide an additional measure of specificity for antisense experiments, at least for targets susceptible to rapid degradation when unprotected. Also, the antisense based methodology described here can be helpful for investigations of bacterial mRNA translation and stability. Nevertheless, the dependence of protein expression on mRNA structure and stability indicate that simple and specific methods for functional studies on the majority of bacterial genes are still lacking. Therefore, we suggest that antisense and other strategies for bacterial gene expression inhibition as a means for gene functional studies [24-26] and drug screening [16,27] should be performed with extra caution with respect to cotranscribed genes.

## Methods

### Bacterial strain and PNAs

*E. coli *strain AS19 was used throughout this study. Due to its hyper permeable nature lower antisense concentrations are required for gene expression inhibition in this strain. PNAs were designed to target the start codon regions of *gfp*, *luc*, *lacZ *and *lacY *(and *lacA*), with the peptide (KFF)_3_K attached for improved cellular uptake [28] and were obtained from Panagene (Daejeon Metropolitan City, Korea). Peptide-PNA sequences are listed in Table 1. Importantly, none of the peptide-PNAs (except anti-*lacA *PNA) displayed any alteration in bacterial growth under the conditions and with the concentrations used in this study.

### Construction of a polycistronic pGRFP-sep plasmid

Cloning vector pGRFP [8] expressing GFP and DsRed as a fusion protein was kindly provided by Stephanie Mohr at Dana-Farber/Harvard Cancer Center DNA Resource Core, US. A GFP stop codon, a *BglII *cleavage site, a Shine-Dalgarno sequence and a unique PNA binding site were introduced between *gfp *and *DsRed *using upstream primer 5'-gcgagatctcatttgtatagttcatccatgccatgtgtaatcccagcagc-3' and downstream primer 5'-tatagatctaaggaggaaacagaatggcctcctccgaggacgtcatca-3'. Cleavage with *BglII *and ligation yielded pGRFPsep, expressing *gfp *and *DsRed *as a single mRNA encoding two different proteins (Figure 1). pGRFPsep was transformed into *E. coli *AS19 where the double reporter system was constitutively expressed.

### Simultaneous assay of GFP and RFP

*E. coli *AS19 and AS19/pGRFPsep were grown for around 12 h in 2 ml Mueller Hinton (MH) broth at 37°C with constant shaking at 225 rpm. Optical density (OD) was measured at 550 nm and the cultures were diluted to 1.25 × 10^6 ^bacteria per ml in MH. Aliquots (80 μl) were added to the wells of a 96 well plate (COSTAR^® ^3474, Corning Inc., NY). Plates were incubated in a VERSAmax spectrophotometer (Molecular Devices Corporation, CA, USA) at 37°C with shaking and OD measurements taken each five minutes. When OD_550 _had increased by ~0.025, dilutions of anti-*gfp *or anti-*DsRed *PNAs were added to make total culture volumes of 100 μl. PNAs directed to *lacZ *and *lacY *mRNA were also included to provide controls. Cultures were harvested in late logarithmic phase after an increase in OD_550 _of ~0.1.

For GFP/DsRed level determinations, AS19 and AS19/pGRFP-sep were lysed by mixing 50 μl cell samples with 50 μl lysis buffer (Pierce Biotechnology, prod. no. 78990) in a 96-well assay plate ("Costar 3632", Corning Inc.). Fluorescence measurements were carried out in a NOVOstar fluorescence/luminescence reader (BMG Labtechnologies, NC, USA). Lysates were excited at 485/560 nm and fluorescence emission was detected at 520/590 nm. Total fluorescence was determined by 100 flashes with maximum capacity of 65000 relative fluorescence units (RFU). Background fluorescence was determined with plasmid free AS19 cell lysates.

### Simultaneous assay of beta-galactosidase, lactose permease and thiogalactoside transacetylase

*E. coli *AS19 cells were pre-grown overnight in 2 ml MH broth at 37°C with constant shaking at 225 rpm. Cultures were diluted to 1.25 × 10^6 ^bacteria per ml in MH and aliquots (200 μl) corresponding to approximately 2.5 × 10^5 ^cells were added to the wells of a 96 well plate. Dilutions of PNAs were added together with IPTG to make a total culture volume of 250 μl with 100 μM IPTG. IPTG- and PNA-free cultures were used as controls. Plates were incubated in a VERSAmax spectrophotometer 37°C with shaking and OD measurements each five minutes until an increase in OD_550 _of ~0.1 was reached. Samples of the cultures were treated as follows:

#### LacZ assay

Samples of *E. coli *cultures (20 μl) were mixed with 20 μl lysis buffer and incubated one hour at room temperature. Beta-galactosidase content of the samples was determined by addition of 160 μl Z-buffer and 20 μl ONPG solution [29], followed by kinetic spectrophotometric measurements of colour conversion at 420 nm every 30 s with 5 s shaking. Curve slopes were used to determine relative levels of beta-galactosidase activity in the samples.

#### LacY assay

Aliquots of 150 μl culture were mixed with 20 μl chloramphenicol to give a final 150 μg/ml antibiotic concentration. Samples were incubated on ice for 10 min and 150 μl were transferred to 96-well plates containing 50 μl ^14^C-lactose (10 μM final concentration). After incubation at 37°C with shaking every five minutes for 30 min, cells were applied to a glass fiber filter (PerkinElmer, prod. no: 1450-441) and washed with PBS in a TOMTEC (Tomtec Inc., Hamden, CT, USA) cell harvester. The filter was dried in a microwave oven, placed in a sample bag (prod. no: 1450-432) together with a Meltilex A melt-on scintilator sheet (prod. no: 1450-441) and sealed in a Heatsealer (PerkinElmer). Lactose permease mediated uptake of ^14^C labelled lactose was detected in a Wallac 1450 MicroBeta^® ^TriLux scintillation counter.

#### LacA assay

Aliquots of 50 μl culture were transferred to 50 μl lysis buffer and incubated at room temperature for 20 minutes. Samples were kept at 70°C for 5 min before a 15 min centrifugation at 20800 g in RT. Supernatants (50 μl) were transferred to 10 μl assay media (5 mg/ml acetyl CoA and 250 mg/ml IPTG in 0.05 M Tris and 0.01 M EDTA, pH 7.9) followed by a one hour incubation at 25°C. Addition of 200 μl DTNB solution (250 μg/ml in 0.05 M Tris pH 7.9) was followed by OD-measurement at 412 nm to determine relative amounts of *lacA *gene product.

Controls and standard curves were provided for all assays using un-induced AS19 cells and/or mixtures of un-induced and induced cells.

An additional control for anti-*lacZ *and anti-*lacY *PNA specificity was performed on AS19 cells carrying plasmid pLux1 [31] that contains the *luxA *and *luxB *genes from *Vibrio harveyi*. Cells were grown under the same conditions as in the *lac*-assays with increasing concentrations (0–1000 nM) of anti-*lacZ *and anti-*lacY *PNA. Cultures were either allowed to grow continuously for generating growth curves or harvested after an increase in OD_550 _of ~0.1 cells for quantification of luciferase expression following a previously described protocol [32]. Plasmid-free AS19 were used to remove background luminescence.

### mRNA quantification by reverse transcription and real time PCR

*E. coli *AS19 treated with different concentrations of anti-*lacZ *or anti-*lacY *PNA in the presence or absence of 100 μM IPTG were incubated until the OD_550 _increased by ~0.1. Cells with equal treatment were pooled to get two separate 1 ml samples for each treatment. Total RNA was extracted with TRIZOL^®^Reagent following the manufacturer's instructions (Invitrogen). Samples were dissolved in 34 μl RNase-free water (Invitrogen) and treated with RNase-free DNase (Sigma Aldrich). RNA concentrations were determined by OD-readings on an Ultrospec 3000 (Pharmacia Biotech, Uppsala, Sweden). Reverse transcription was performed using random hexamers with 1 μg RNA in 50 μl reactions following the manufacturers protocol (Applied Biosystems). Aliquots derived from 50 ng RNA were analyzed by qPCR on an ABI PRISM 7000 (Applied Biosystems) according to the manufacturers protocol. Samples were run in triplicates and the data obtained were analyzed with ABI Prism SDS Software (Applied Biosystems). The ΔΔCt method was used to calculate *lacZ *and *lacY *gene expression using rRNA as an internal standard [30]. Sequences for the *lacZ *and *lacY *primers were selected to amplify regions near the 5' end of the coding regions to increase the chances of detecting functional transcripts as *lac *mRNAs are degraded in a 5' to 3' direction [20,33], and were: *lacZ *forward 5'-ccctggcgttacccaacttaa-3'; *lacZ *reverse 5'-gcgggcctcttcgctattac-3'; *lacY *forward 5'-cgcaaatacctgctgtggatta-3' and *lacY *reverse 5'-tggcccgaagataaaaataaagaa-3'. The primer binding sites did not interfere with antisense PNA target sites (see Figure 3A). Primer sequences for rRNA were taken from [34] and were: rRNA forward 5'-catgccgcgtgtatgaagaa-3' and rRNA reverse 5'-cgggtaacgtcaatgagcaaa-3'. RNA isolation and quantification was repeated at four different occasions giving eight samples and 24 data points per PNA concentration. Data comparisons were made to a normalised control value obtained by calculating the mean at each experimental occasion. Statistical calculations were performed in Excel using unpaired t-tests assuming equal variance among groups and data are presented as the mean with error bars reflecting the standard deviation. Significance was set at p < 0.05.

### Parallel analysis of LacZ activity and *lacZ *mRNA abundance

*E. coli *AS19 treated with anti-*lacZ *PNA concentrations ranging from 0 to 400 nM at 50 nM intervals were grown in six double replicas in the presence or absence of 100 μM IPTG. Cultures were incubated under the same conditions as described above and harvested when OD_550 _had increased by ~0.1. Samples of 20 μl were removed from each well to be used for separate LacZ activity measurements as described above, and the remainder of the cultures were pooled into groups with equal treatment. Total RNA was extracted and treated with DNase I using RiboPure Bacteria kit (Ambion), according to the manufacturer's protocol. Quantification of *lacZ *mRNA was performed as described above. Mean values from the six replicates of each treatment were used to determine relative LacZ activity and the averages of three replicates derived from each sample pool were used to estimate mRNA abundance. The experimental procedure was repeated at three different occasions, and mean values with error bars were plotted as a histogram. Regression analysis was conducted using VassarStats [35].

## Authors' contributions

RD designed, carried out and analysed most experiments under the supervision of LG during and after his PhD. AN conducted experiments that contributed to the reporter gene system used to study the expression of both ORFs within an operon. AK assisted with experiments to establish the gene expression assays and helped with data analysis and interpretation. SG carried out the detailed dose response experiment involving simultaneous analysis of LacZ protein activity and *lacZ *mRNA abundance. RD drafted the manuscript in collaboration with LG. All authors read and approved the final manuscript.

## Supplementary Material

Additional file 1 **Effects of anti-lacZ and anti-lacY PNAs on growth and Lux-expression**. Bacterial growth was monitored over time as culture turbidity (OD_550_) after treatment with 0 (circles), 100 (squares), 250 (diamonds), 500 (x-marks), 750 (+-marks) and 1000 (triangles) nM concentrations of either anti-*lacZ *PNA (**A**) or anti-*lacY *PNA (**B**). (**C**) Expression of a bacterial luciferase reporter gene (*lux*) is shown as relative units normalised to growth after treatment with increasing concentrations of either anti-*lacZ *PNA (squares) or anti-*lacY *PNA (triangles). Background luminescence was removed using pLux1-free cells. All curves illustrate mean values from five replicates +/- std for Lux values. Click here for file

## Figures and Tables

**Table 1:** PNA structures and mRNA target sites

PNA	Structure	Target site
anti-*gfp*I	(KFF)3K-eg1-aataacctcc	-11 to -2
anti-*gfp*II	(KFF)3K-eg1-cgcataaata	-5 to +5
anti-*DsRed*I	(KFF)3K-eg1-attctgtttcc	-9 to +2
anti-*DsRed*II	(KFF)3K-eg1-ccattctgtt	-6 to +4
anti-*lacZ*	(KFF)3K-eg1-catagctgtttc	-9 to +3
anti-*lacY*	(KFF)3K-eg1-cataatggattt	-9 to +3
anti-*lacA*	(KFF)3K-eg1-caatgcgatc	-7 to +3

mRNA target sites are shown relative to translation start sites of the respective coding sequences.
