# Supplementary material for: Variable coordination of cotranscribed genes in Escherichia coli following antisense repression
Source: BMC Microbiol. 2006 Nov 21;6:97. doi: 10.1186/1471-2180-6-97 (PMC1661596; doi:10.1186/1471-2180-6-97)
Supplement: Additional file 1 — Effects of anti-lacZ and anti-lacY PNAs on growth and Lux-expression. Bacterial growth was monitored over time as culture turbidity (OD550) after treatment with 0 (circles), 100 (squares), 250 (diamonds), 500 (x-marks), 750 (+-marks) and 1000 (triangles) nM concentrations of either anti-lacZ PNA (A) or anti-lacY PNA (B). (C) Expression of a bacterial luciferase reporter gene (lux) is shown as relative units normalised to growth after treatment with increasing concentrations of either anti-lacZ PNA (squares) or anti-lacY PNA (triangles). Background luminescence was removed using pLux1-free cells. All curves illustrate mean values from five replicates +/- std for Lux values. [file 1471-2180-6-97-S1.pdf]

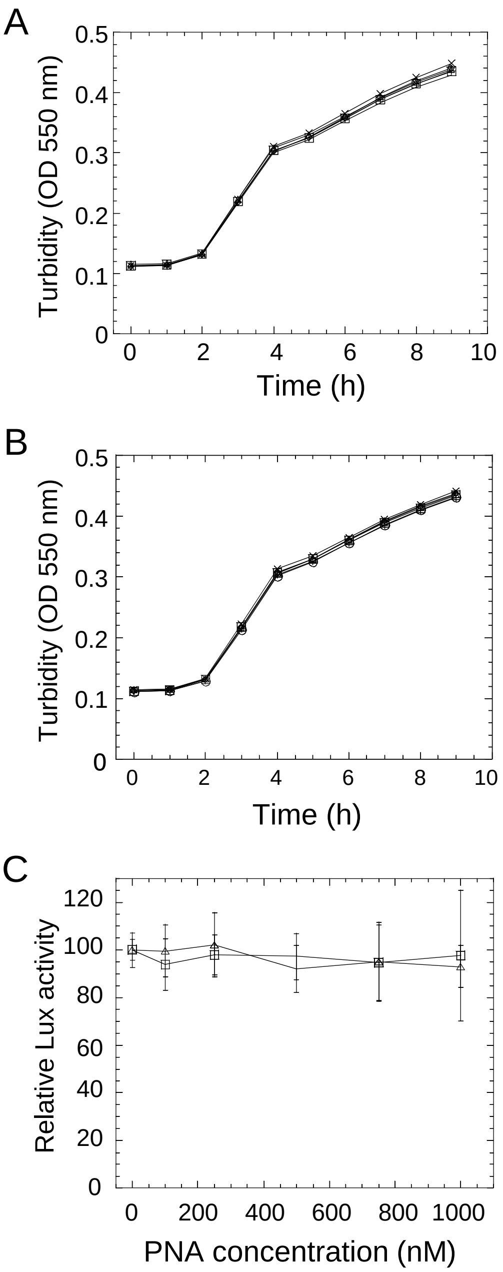

Additional file 1 - Effects of anti-*lacZ* and anti-*lacY* PNAs on growth and Lux-expression.

Bacterial growth was monitored over time as culture turbidity (OD_{550}) after treatment with 0 (circles), 100 (squares), 250 (diamonds), 500 (x-marks), 750 (+-marks) and 1000 (triangles) nM concentrations of either anti-*lacZ* PNA (**A**) or anti-*lacY* PNA (**B**). (**C**) Expression of a bacterial luciferase reporter gene (*lux*) is shown as relative units normalised to growth after treatment with increasing concentrations of either anti-*lacZ* PNA (squares) or anti-*lacY* PNA (triangles). Background luminescence was removed using pLux1-free cells. All curves illustrate mean values from five replicates +/- std for Lux values.
